# Supplementary material for: Rapid purification of brain protein complexes containing active and inactive forms of the G protein Gαo
Source: PLoS One. 2025 Dec 4;20(12):e0337998. doi: 10.1371/journal.pone.0337998 (PMC12677461; doi:10.1371/journal.pone.0337998)
Supplement: S1 Table — (DOCX) [file pone.0337998.s001.docx]

# Appendix A. Supporting Information

**Table S1: Key Reagents**

| **Chemical Name** | **Source** | **Catalogue No.** |
| --- | --- | --- |
| NuPAGE™ Bis-Tris Mini Protein Gels | Thermo Scientific | NP0321BOX |
| Bradford Reagent | Bio-Rad | 5000006 |
| GDP | Sigma Aldrich | G7127 |
| GTPγS | Abcam | ab146662 |
|  | BIOLOG, Life Science Institute | G019 |
| Goat anti mouse-HRP | Bio-Rad | 1706516 |
| Goat anti Rabbit-HRP | Bio-Rad | 1706515 |
| Rabbit anti-Sheep-HRP | Thermo Scientific | 61-8620 |
| LDS loading buffer | Thermo Scientific | NP0007 |
| Pierce™ Crosslink IP Kit | Thermo Scientific | 26147 |
| Imperial Protein Stain | Thermo Scientific | 24615 |
| NuPAGE MOPS SDS Running Buffer | Thermo Scientific | NP0001 |
| Mouse monoclonal anti- Gα_o_ antibody | Santa Cruz | sc-13532 |
| Mouse monoclonal anti-Gβ antibody | Santa Cruz | sc-166123 |
| PBS (10X), pH 7.4 | Thermo Scientific | 70011044 |
| Page Ruler plus Protein Ladder | Thermo Scientific | 26619 |
| SuperSignal™ West Pico PLUS Chemiluminescent Substrate | Thermo Scientific | 34579 |
| Super Signal West Femto Maximum Sensitivity substrate | Thermo Scientific | 34094 |
| Superose^TM^ 6 10/300 GL column | Cytiva | 17517201 |
