# Supplementary material for: Rapid purification of brain protein complexes containing active and inactive forms of the G protein Gαo
Source: PLoS One. 2025 Dec 4;20(12):e0337998. doi: 10.1371/journal.pone.0337998 (PMC12677461; doi:10.1371/journal.pone.0337998)

Related to Fig 1B

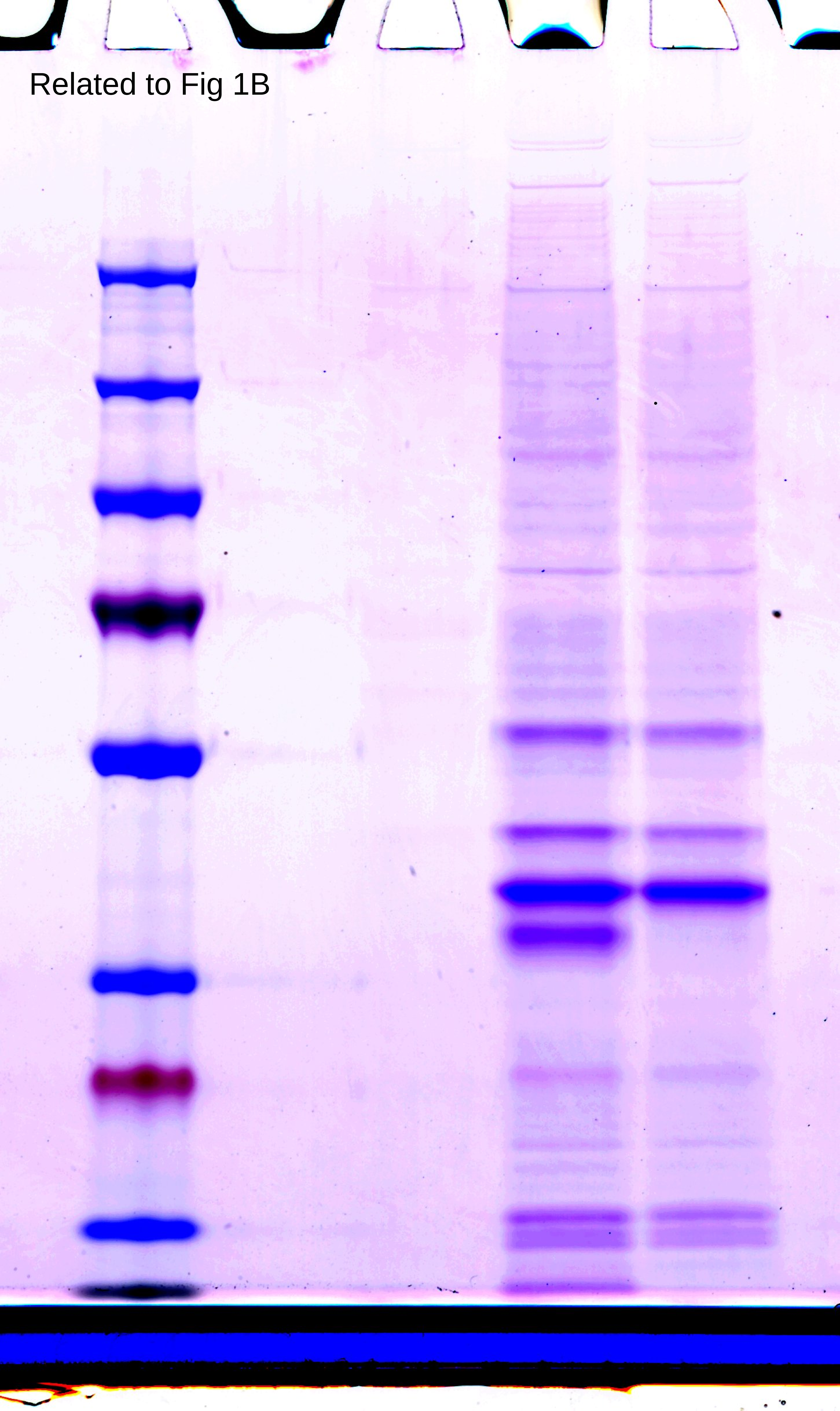

Related to Fig1C top blot

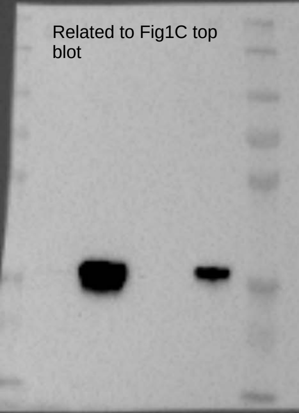

Related to Fig1C bottom blot

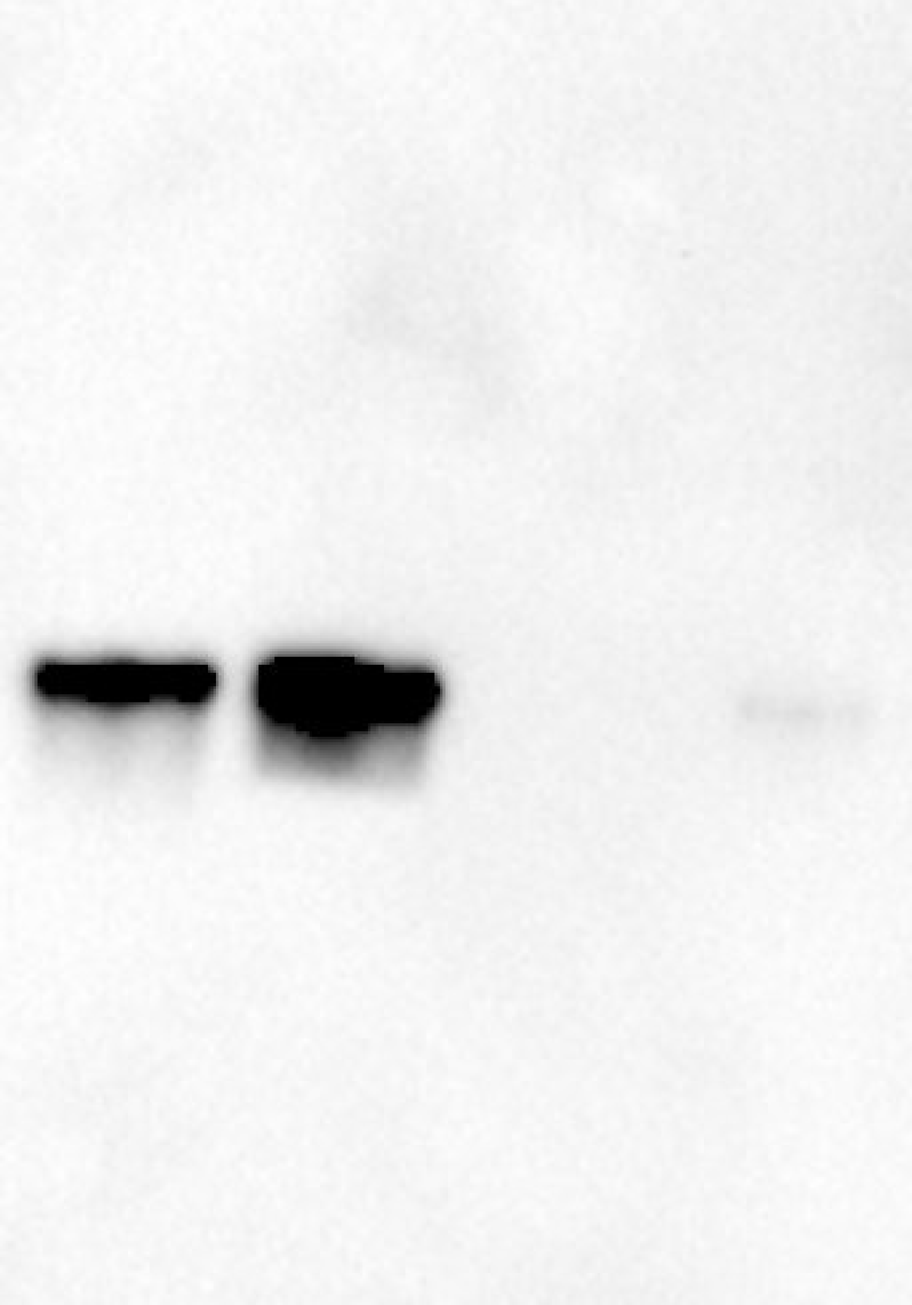

Related to Fig1E, top

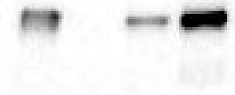

Related to Fig 1E, bottom

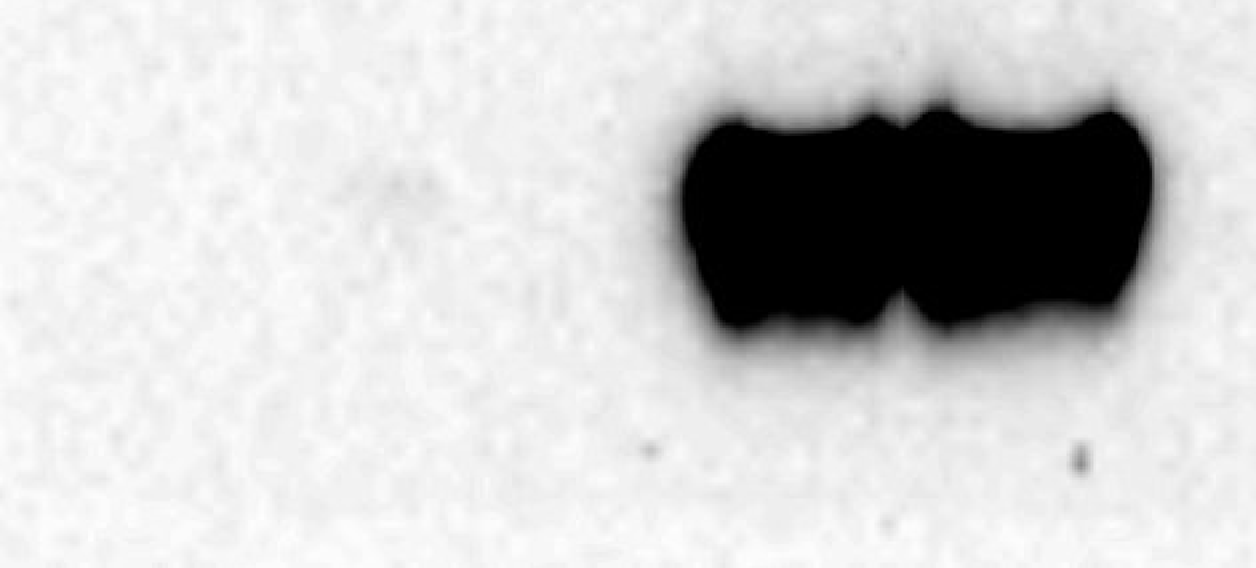

Fig2C, G alpha blot

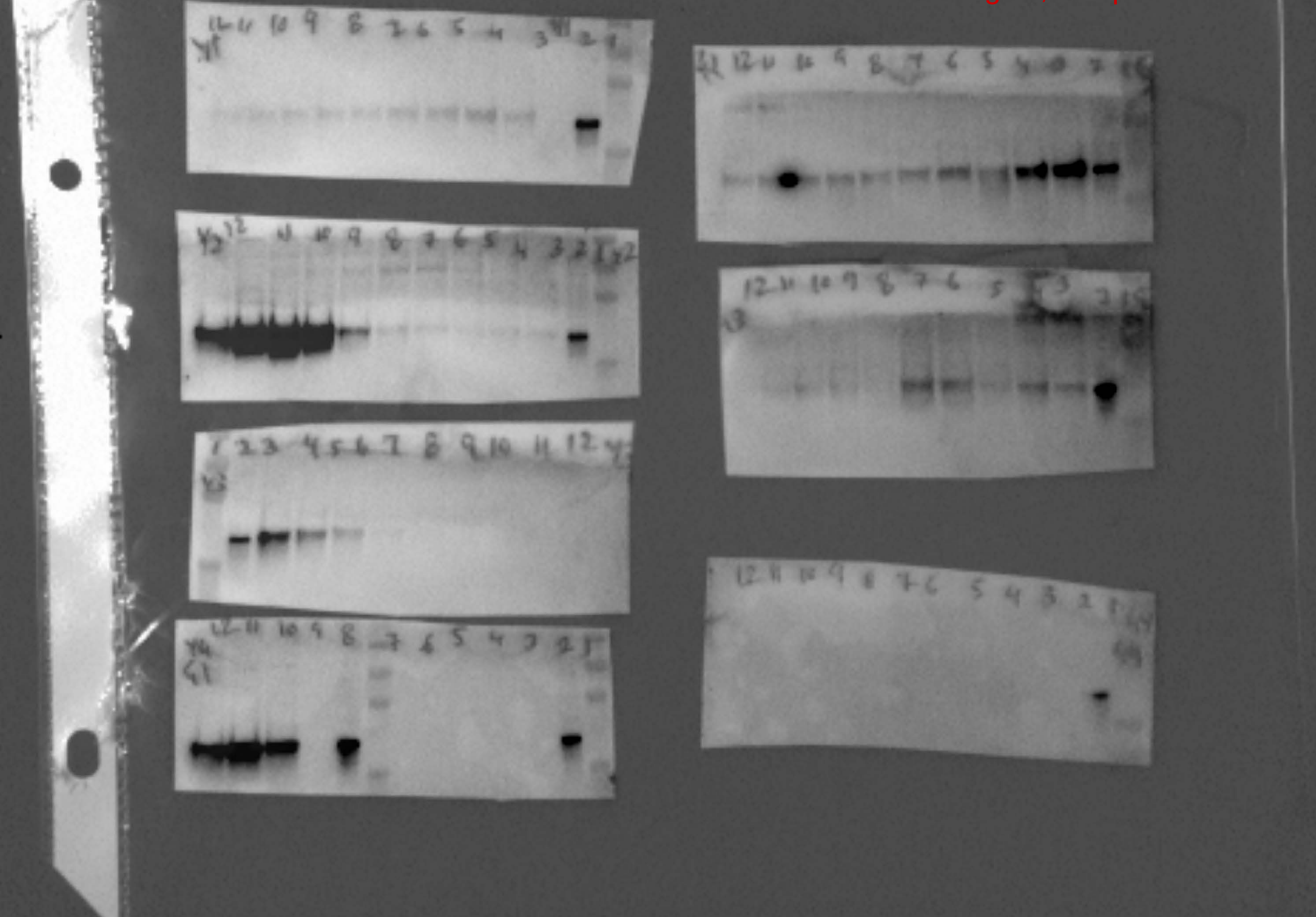

Fig 2C, G beta blot

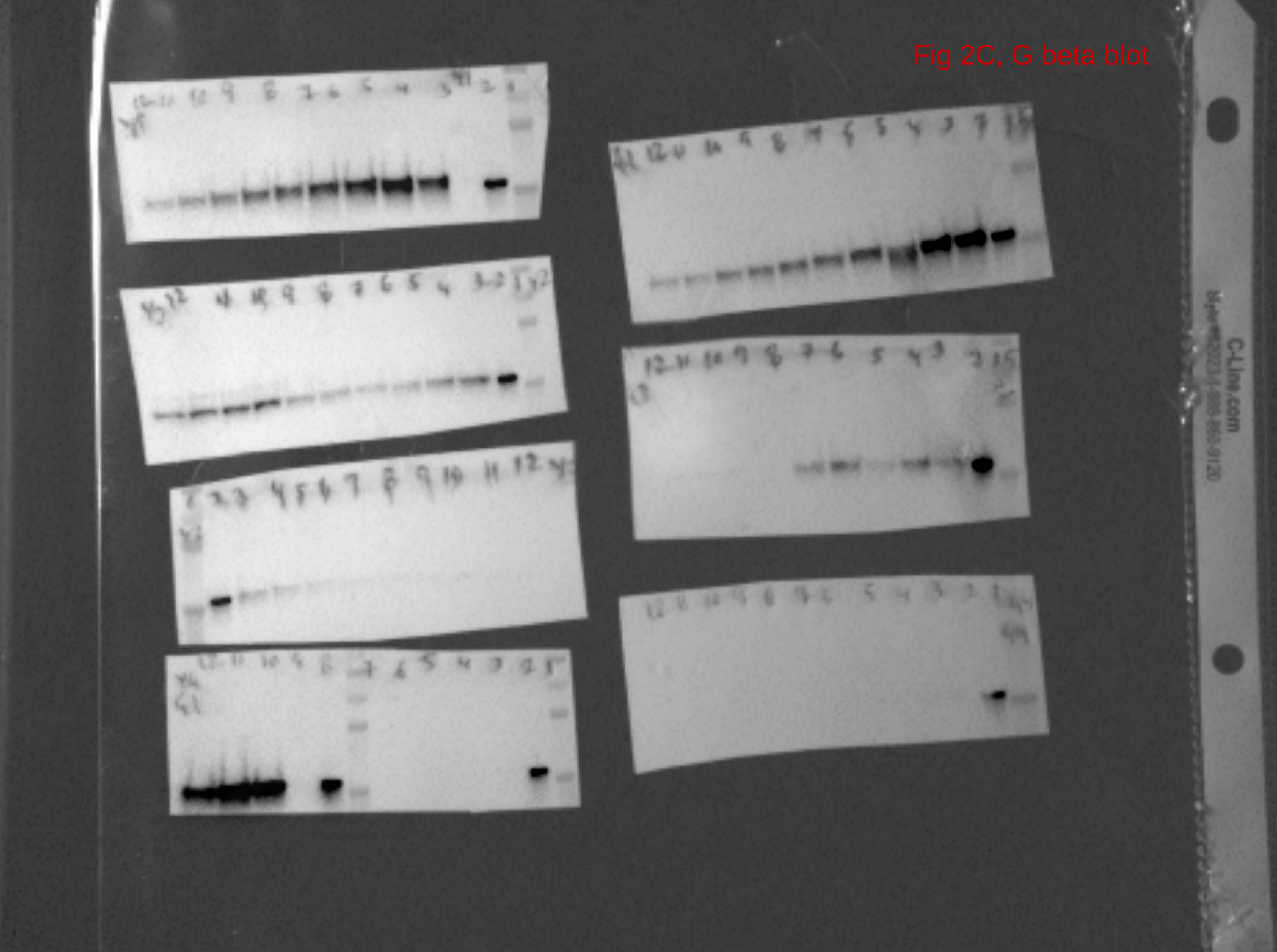

Supplement: S1 File — (PDF) [file pone.0337998.s002.pdf]
