# Supplementary figures and images for: Rapid purification of brain protein complexes containing active and inactive forms of the G protein Gαo
Source: PLoS One. 2025 Dec 4;20(12):e0337998. doi: 10.1371/journal.pone.0337998 (PMC12677461; doi:10.1371/journal.pone.0337998)

S1 Figure

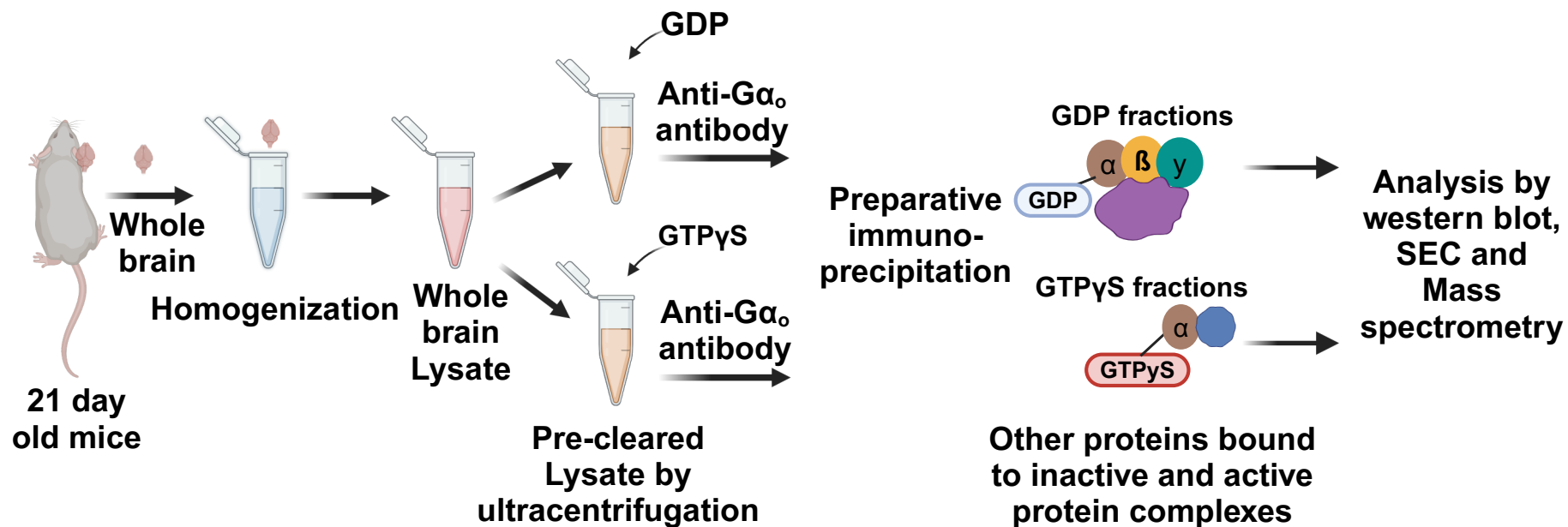

Supplement: S1 Figure — (PDF) [file pone.0337998.s003.pdf]
