# Supplementary material for: Rapid purification of brain protein complexes containing active and inactive forms of the G protein Gαo
Source: PLoS One. 2025 Dec 4;20(12):e0337998. doi: 10.1371/journal.pone.0337998 (PMC12677461; doi:10.1371/journal.pone.0337998)
Supplement: Attachment — Submitted filename: Review_19360.docx [file pone.0337998.s004.docx]

The authors have done a beautiful job of purifying complicated Gαo protein from mouse brain lysate and tried to identify its binding to effectors in the manuscript. They have given a detailed introduction to the protein of interest and a detailed methodology of their experiments. Though the study in the manuscript is incomplete and inconclusive, it forms a base for future studies with these proteins.

I found the manuscript interesting, and it also projects interesting work. Having said that, there are some concerns about the manuscript, which would enhance the manuscript and help in future research directions even better. Below are my comments on the manuscript:

1. In the introduction part, it would help to provide some information about the effectors of Gαo proteins. The authors have abruptly jumped into discussing effector proteins.
2. The authors have mentioned SNARE proteins. Nowhere in the manuscript have they described the abbreviation and its significance.
3. The authors say they prepared brain lysates multiple times to get 12 mg/ml. The scientific way of saying this would be to quantitate it by saying how many brain samples.
4. There are a couple of abbreviations for IP. The authors should either use the full form for one of them or should name the buffer with some other acronym.
5. The manuscript in general has errors/typos that need to be corrected. Also, the units should be written in a consistent manner throughout the manuscript like μL, mM, μM etc.
6. For SEC, the authors have mentioned the specific fraction groups collected but never mentioned the rationale of it. The better way of saying this would be all the fractions were collected.
7. The authors have mentioned GTPγS as a non-hydrolysable analog of GTP. However, it is a slowly hydrolyzing analog. For this study, better suited would have been other non-hydrolysable analogs such as GMPPCP/GMPPNP.
8. An alternative method to check the loading could have been HPLC, which would be more quantitative and unbiased.
9. In the western blot of fig. 1, the loading controls are absent, which is standard for the blot images.
10. In the blot of fig 1C, the Gαo bands should be identical, as per the hypothesis and explanations in the text. Nothing has been discussed why do you see a difference in intensity of Gαo in the GDP and GTPγS blots.
11. The author say mass spectrometry could be used to check the effector-binding. A lane in the SDS-PAGE with crude extract, which is not modified to load any nucleotide, would be an interesting sample to compare.
12. In fig.2, it is interesting and confusing at the same time, as to how there is a huge fraction from 37-45 which absorbs at 280, but does not read in Coomassie. It would be of interest to get the MALDI done for these samples and check the possibility. That may also give a conclusion to the manuscript.
