# Supplementary material for: Rapid purification of brain protein complexes containing active and inactive forms of the G protein Gαo
Source: PLoS One. 2025 Dec 4;20(12):e0337998. doi: 10.1371/journal.pone.0337998 (PMC12677461; doi:10.1371/journal.pone.0337998)
Supplement: Attachment — Submitted filename: Response to Reviewers.docx [file pone.0337998.s005.docx]

**Manuscript ID:** PONE-D-25-19360

**Manuscript Title**: Rapid purification of brain protein complexes containing active and inactive forms of the G protein Gα_o_

Dear Dr. Ajit,

We thank you for your email dated June 19^th^, 2025, and for the opportunity to revise and resubmit our manuscript entitled, “*Rapid purification of brain protein complexes containing active and inactive forms of the G protein Gα_o_*” **(PONE-D-25-19360).**

We are grateful to you and the reviewers for the insightful and constructive comments, which have helped us to significantly improve the clarity and quality of our manuscript. We have carefully considered all points raised and have revised the manuscript accordingly (with track changes). Please find below our detailed responses to each of the editor’s and reviewers’ comments.

Please note that editor’s and reviewers' comments are presented in **blue font**, while our responses are provided in **standard black font.** Corresponding changes about the reviewers' comments, along with the line numbers mentioned in the response, have been highlighted in **red font** in the main revised manuscript.

Please note that the corrected line numbers mentioned in our responses here correspond to the lines marked using the "**Simple Markup"** view under the **"Review"** tab in the revised manuscript. This helps maintain readability while still indicating where revisions have occurred. If this setting is not enabled, you can simply go to the Review tab, locate the Markup section, and in the first drop-down, **Display for Review,** select **“Simple Markup”** (for Microsoft Word 2021).

**Response to #Editor’s comments:**

1. When submitting your revision, we need you to address these additional requirements.

Please ensure that your manuscript meets PLOS ONE's style requirements, including those for file naming. The PLOS ONE style templates can be found at https://journals.plos.org/plosone/s/file?id=wjVg/PLOSOne_formatting_sample_main_body.pdf and <https://journals.plos.org/plosone/s/file?id=ba62/PLOSOne_formatting_sample_title_authors_affiliations.pdf>

**Response:** We thank the editor for providing us with the templates. We have referred to them and formatted the main text and affiliations according to the provided templates in the revised version of the manuscript.

2. To comply with PLOS ONE submissions requirements, in your Methods section, please provide additional information regarding the experiments involving animals and ensure you have included details on (a) methods of sacrifice, (b) methods of anaesthesia and/or analgesia, and (c) efforts to alleviate suffering.

**Response:** We thank the editor for highlighting this important requirement. We recognize the importance of providing clear and complete ethical information following PLOS ONE’s submission guidelines for animal research. We have added all the required information in the Materials and Methods Section.

**Changes made in the manuscript:** Added a sub-section in the Materials and Methods Section, “Animal Handling and Ethics Statement”. Added lines 126 to 140.

3. Thank you for stating in your Funding Statement:
   S.K.'s laboratory is funded by Department of Biotechnology (DBT), Ministry of Science and Technology India (BT/PR38584/MED/122/247/2020), Department of Science and Technology (DST), Ministry of Science and Technology India (CRG/2021/000732) and DBT/Wellcome Trust India Alliance (IA/I/22/2/506480). M.R.K. is supported by NIH grant R01NS086932.

Please provide an amended statement that declares *all* the funding or sources of support (whether external or internal to your organization) received during this study, as detailed online in our guide for authors at <http://journals.plos.org/plosone/s/submit-now>. Please also include the statement “There was no additional external funding received for this study.” in your updated Funding Statement. Please include your amended Funding Statement within your cover letter. We will change the online submission form on your behalf."

**Response:** We thank the editor for letting us know. We have provided the amended statement regarding the funding statement in the cover letter. Kindly include it in the manuscript from your end.

4. Thank you for stating the following in the Acknowledgments Section of your manuscript:
   We thank Dr Prasad Abnave (NCCS, Pune) for critical evaluation and valuable feedback on the manuscript. We heartily thank our lab members, Ajay Pradhan, Vandan Maurya, and Debolina Sarkar, for their continuous and generous support, and Nikhat Khan for skillful technical assistance.
   S.K.’s laboratory is funded by Department of Biotechnology (DBT), Ministry of Science and Technology India (BT/PR38584/MED/122/247/2020), Department of Science and Technology (DST), Ministry of Science and Technology India (CRG/2021/000732) and DBT/Wellcome Trust India Alliance (IA/I/22/2/506480). M.R.K. is supported by NIH grant R01NS086932.

We note that you have provided funding information that is not currently declared in your Funding Statement. However, funding information should not appear in the Acknowledgments section or other areas of your manuscript. We will only publish funding information present in the Funding Statement section of the online submission form.
Please remove any funding-related text from the manuscript and let us know how you would like to update your Funding Statement. Currently, your Funding Statement reads as follows:
S.K.’s laboratory is funded by Department of Biotechnology (DBT), Ministry of Science and Technology India (BT/PR38584/MED/122/247/2020), Department of Science and Technology (DST), Ministry of Science and Technology India (CRG/2021/000732) and DBT/Wellcome Trust India Alliance (IA/I/22/2/506480). M.R.K. is supported by NIH grant R01NS086932.

Please include your amended statements within your cover letter; we will change the online submission form on your behalf.

**Response:** We thank the editor for informing us. We have provided the amended statement regarding the Acknowledgements in the cover letter. Kindly include it in the manuscript from your end.

5. PLOS requires an ORCID iD for the corresponding author in Editorial Manager on papers submitted after December 6th, 2016. Please ensure that you have an ORCID iD and that it is validated in Editorial Manager. To do this, go to 'Update my Information’ (in the upper left-hand corner of the main menu), and click on the Fetch/Validate link next to the ORCID field. This will take you to the ORCID site and allow you to create a new iD or authenticate a pre-existing iD in Editorial Manager.

**Response:** We thank the editor for letting us know. We have validated our ORCID ID as per the given instructions.

6. Please include your full ethics statement in the ‘Methods’ section of your manuscript file. In your statement, please include the full name of the IRB or ethics committee that approved or waived your study, as well as whether or not you obtained informed written or verbal consent. If consent was waived for your study, please include this information in your statement as well.

**Response**: We thank the editor for bringing this point to our attention. We have included all the necessary information in the Methods Section.

**Changes made in the manuscript:** Added a sub-section in the Materials and Methods Section, “Animal Handling and Ethics Statement”. Added lines 126 to 140.

7. We notice that your supplementary table is included in the manuscript file. Please remove and upload with the file type ‘Supporting Information’. Please ensure that each Supporting Information file has a legend listed in the manuscript after the references list.

**Response:** We have removed the table from the manuscript and re-uploaded it with the file type “Supporting Information”. We have listed a legend for the supporting information after the References.

**Changes made in the manuscript:** Added lines 494 to 496 and uploaded supporting information

8. Please include captions for your Supporting Information files at the end of your manuscript, and update any in-text citations to match accordingly. Please see our Supporting Information guidelines for more information (<http://journals.plos.org/plosone/s/supporting-information>)

**Response:** We have made the necessary additions and provided in-text citations.

.

9. PLOS ONE now requires that authors provide the original uncropped and unadjusted images underlying all blot or gel results reported in a submission’s Figures or Supporting Information files. This policy and the journal’s other requirements for blot/gel reporting and figure preparation are described in detail at https://journals.plos.org/plosone/s/figures#loc-blot-and-gel-reporting-requirements and <https://journals.plos.org/plosone/s/figures#loc-preparing-figures-from-image-files>. When you submit your revised manuscript, please ensure that your figures adhere fully to these guidelines and provide the original underlying images for all blot or gel data reported in your submission. See the following link for instructions on providing the original image data: <https://journals.plos.org/plosone/s/figures#loc-original-images-for-blots-and-gels>. In your cover letter, please note whether your blot/gel image data are in Supporting Information or posted at a public data repository, provide the repository URL if relevant, and provide specific details as to which raw blot/gel images, if any, are not available. Email us at plosone@plos.org if you have any questions.

**Response:**

We thank the editor for letting us know. As per the journal’s requirement, we have uploaded all the raw blot and gel images as a separate file. We have also acknowledged the same in the cover letter.

**Response to Reviewer #1 comments:**

*The manuscript authored by Yadav et al., entitled "Rapid purification of brain protein complexes containing active and inactive forms of the G protein Gα_o_" presents a study aimed at identifying downstream effectors of the Gα_o_ protein, the most abundant neuronal heterotrimeric G protein. The authors use a novel biochemical approach to isolate native Gα_o_-GTP protein complexes from mouse brain lysates. Their findings rely on immunoprecipitation and size exclusion chromatography to analyze Gα_o_ complexes, demonstrating enrichment in Gα_o_ and depletion of other GTPYs. This study addresses a significant gap in understanding Gα_o_ signaling, as no direct effectors have been well-characterized despite its abundance and major role in neuronal function. The methods are novel and well-executed, but several areas require clarification, additional controls, and improved discussion to strengthen the manuscript’s impact and rigor. My specific comments are as follows:*

**Response:** We thank the reviewer for the critical evaluation and the constructive feedback provided for the manuscript. The suggestions have certainly helped us to increase the quality and clarity of the manuscript. Here, we provide a point-by-point response to each of the reviewer’s comments.

Please note that the corrected line numbers mentioned in our responses here correspond to the lines marked using the "**Simple Markup"** view under the **"Review"** tab in the revised manuscript. This helps maintain readability while still indicating where revisions have occurred. If this setting is not enabled, you can simply go to the Review tab, locate the Markup section, and in the first drop-down, **Display for Review,** select **“Simple Markup”** (for Microsoft Word 2021).

1. *The introduction mentions genetic and interaction screens that failed to identify Gα_o_ effectors, but does not compare the current method’s advantages over these approaches in detail. A stronger rationale for why biochemical purification will likely succeed where genetic screens failed would strengthen the manuscript. Expand the introduction to discuss the limitations of yeast two-hybrid and genetic screens more explicitly and highlight how the current method overcomes these.*

**Response:** We thank the reviewer for this thoughtful and constructive comment. You're right that a more explicit comparison between classical genetic/interaction screens and our biochemical purification approach would enhance the introduction. As per your suggestion, we have also added information that explicitly explains studies done in yeast and *C. elegans* **(Revised manuscript Lines 80 to 89)***.* We have discussed the limitations of genetic screens **(Revised manuscript Lines 94 to 97)** and Y2H interaction systems in the revised version of the manuscript. **(Revised manuscript, Lines 101-109)**. We have also added a paragraph that highlights how our method overcomes those limitations. **(Revised manuscript, Lines 112 to 119).**

**Changes made in the manuscript:** Added lines 80 to 89 (studies done in yeast and *C. elegans*) and references [17] and [18], with subsequent changes in reference numbers. Added lines 94 to 97 (Limitations of genetic screens), 101 to 109 (Limitations of Y2H systems), and 112 to 119 (Advantages of our method).

2. *The manuscript aims to identify Gα_o_ effectors but does not report any specific effector proteins identified through the described methods. The discussion mentions that effectors may be present at low abundance and detectable by mass spectrometry, but no such data are provided. This is a major limitation, as the study’s primary goal appears unfulfilled. If mass spectrometry was performed, include preliminary results or clarify why such data are not presented. If not performed, explicitly state this as a limitation and outline plans for future work to identify effectors using these methods.*

**Response:** We thank the reviewer for raising this crucial point. Our goal was first to establish a reliable and reproducible protocol that could distinguish active Gα_o_-GTP complexes from the inactive Gα_o_-GDP state and thereby enable identification of direct Gα_o_ effectors. Now that this method has been validated and optimized, we have validated a known effector of Gα_o,_ GPRIN-1_,_ performed mass spectrometry, and incorporated preliminary results into the revised version, as well as revised Fig. 1.

**Changes made in the manuscript:** Added lines 195 to 197 (Methods-information about anti-GPRIN-1 antibody), Lines 216-235 (Methods- Mass spectrometry protocol), Lines 289 to 307 (Results for GPRIN-1 western blot and mass spectrometry). Also added revised Fig.1 Panel E and G. Added lines 469 to 471 (figure legend- Revised Fig. 1, Panel E), and lines 472 to 476(figure legend- Revised Fig. 1, Panel G).

3. *The observed molecular weights of Gα_o_ complexes (1249–945 kDa for GDP-bound and 98–398 kDa for GTPγS-bound) are much larger than expected, even accounting for Triton X-100 micelles. The manuscript attributes this to potential effector interactions but does not adequately discuss alternative explanations, such as non-specific protein aggregation or micelle size variability. Include controls to rule out non-specific interactions, such as comparing Gα_o_ complexes to those of other G proteins (e.g., Gaq or Gai) under identical conditions. Discuss the impact of detergent micelles on apparent molecular weight more thoroughly, potentially referencing studies on membrane protein complexes in similar systems.*

**Response:** We thank the reviewer for bringing this point to our attention. The apparent molecular weights of Gα_o_ protein complexes observed in our size exclusion chromatography (SEC) experiments are markedly higher than the expected sizes for the Gα_o_ monomer or Gα_o_-βγ heterotrimer. While we interpret this shift as binding of unknown effectors upon Gα_o_ activation, we have also discussed that these elevated values likely also reflect the contribution of detergent micelles, which are known to influence the hydrodynamic behaviour of solubilized membrane proteins. As per your suggestion, we have also added alternative explanations that could be potentially responsible for the change in molecular weights of the complexes.

**Changes made in the manuscript:** Added lines 329 to 332.

4. *Validate anti-Gα_o_ antibody specificity by testing it against lysates from Gao knockout mice or performing co-immunoprecipitation with other G protein subunits (e.g., Gaq or Gai) to confirm selectivity.*

**Response:** We thank the reviewer for this important comment. We agree that validating the specificity of the anti-Gα_o_ antibody is critical to ensuring the reliability of our immunoprecipitation results. The antibody used in this study (Santa Cruz Biotechnology, sc-13532) is a commercially available monoclonal antibody that has been extensively validated by the supplier for use in western blotting and immunoprecipitation of Gα_o_ across multiple species, including mouse. It has also been used experimentally and cited in around 42 publications worldwide. (https://www.scbt.com/p/galpha-o-antibody-a2?srsltid=AfmBOooiqSGcI4Z7TAngA86AAkNsMneroEg3rbS_rzoM7HCPoX5DZ6Yp#citations).

However, to address this concern, we assessed the specificity of the anti-Gα_o_ antibody by analyzing mass spectrometry data for unique peptide spectra corresponding to different Gα subunits. We identified 69 unique peptides specific to Gα_o_, while no unique peptides were detected for Gα_q_ or Gα_i_, supporting the antibody’s selectivity for Gα_o_. Additionally, to address the issue deeply, we performed additional experiments using GPRIN-1, which is an established and specific effector of Gα_o_, as a positive control for interaction. As shown in Revised Fig. 1 Panel E, GPRIN1 strongly co-immunoprecipitates with Gα_o_ in the presence of GTPγS, but not in GDP or control conditions, demonstrating the expected state-dependent interaction. This indicates that the antibody effectively captures Gα_o_ in its active conformation and does not non-specifically pull-down unrelated proteins. Importantly, no GPRIN1 was detected in control IPs, further ruling out non-specific interactions. (Fig. 1E, lower blot).

**Changes made in the manuscript:** Lines 289 to 307 (Results for GPRIN-1 western blot and mass spectrometry) and added panels E, F, and G in revised Fig.1. Added lines 469 to 471 (figure legend- Revised Fig. 1, Panel E), lines 471 to 472 (figure legend- Revised Fig. 1, Panel F) and lines 472 to 476 (figure legend- Revised Fig. 1, Panel G).

5. *The manuscript does not mention the number of biological or technical replicates for key experiments (e.g., immunoprecipitation, size exclusion chromatography). The statement “we prepared brain lysates multiple times” is vague. Specify the number of replicates and include statistically analyzable data where applicable (e.g., for protein yield or band intensity in western blots). Provide error bars or variability measures for quantitative data.*

**Response**: We thank the reviewer for pointing this out. In the revised manuscript, we have mentioned a line above the materials and methods section that states each experiment was performed twice.

**Changes made in the manuscript:** Added lines 123 and 124 (referring to all the experiments) and lines 153 and 154 (referring to preparation of MBL experiments).

6. *Figures 1D and 1E are referenced extensively, but the manuscript does not clearly describe the expected molecular weights of Gβ and Gγ bands. The text mentions Gβ at 32 kDa and Gγ at 10 kDa, but Gγ is not quantified. Annotate figures with molecular weight markers and clarify Gβ band identification in the text*.

**Response:**  We are sorry for not describing the molecular weights of Gβ and Gγ, respectively. In the revised manuscript, we have annotated the correct molecular weights of Gα, Gβ, and Gγ, respectively, in revised Fig. 1 B.

**Changes made in the manuscript:** Provided Revised Fig. 1 B with annotations.

7. *The term “GTPγS” is used in the abstract and early section, while “GTP S” is used later. This inconsistency may confuse readers. Standardize to “GTP S” throughout the manuscript.*

**Response:** We are sorry for the inconsistent use of the terms. We have standardized the use of GTPγS in the manuscript.

**Changes made in the manuscript:** GTPγS was changed to GTPγS wherever applicable.

8. *The manuscript presents a technically sound and novel approach to studying Gα_o_ signaling by isolating native Gα_o_-GTP complexes from mouse brain lysates. The methods are well-executed and have the potential to advance the field, particularly if paired with downstream analyses like mass spectrometry. However, a lack of effector identification, incomplete validation of the antibody specificity, and limited discussion of negative results weaken the current version. Addressing major concerns, particularly providing effector data or clarifying its absence, incorporating the minor suggestions will significantly strengthen the manuscript. I recommend major revisions before publication, focusing on clarifying the results, adding control and enhancing the discussion.*

**Response:** We thank the reviewer for their thoughtful and constructive feedback. In response, we have made substantial revisions to the manuscript. Specifically, we have now included effector identification data using mass spectrometry, validated the antibody specificity and a known effector GPRIN-1 and expanded the discussion accordingly. We believe these additions address the major concerns raised and significantly strengthen the manuscript. We appreciate the reviewer’s insights, which have helped us improve the quality and clarity of our work.

**Changes made in the manuscript:** Added lines 195 to 197 (Methods-information about anti-GPRIN-1 antibody), Lines 216-235 (Methods- Mass spectrometry protocol), Lines 289 to 307 (Results for GPRIN-1 western blot and mass spectrometry. Added revised Fig.1 Panel E and G and added lines 469 to 471 (figure legend- Revised Fig. 1, Panel E) and lines 472 to 476 (figure legend- Revised Fig. 1, Panel G)

**Response to Reviewer #2 comments:**

*The authors have done a beautiful job of purifying the complicated Gαo protein from mouse brain lysate and tried to identify its binding to effectors in the manuscript. They have given a detailed introduction to the protein of interest and a detailed methodology of their experiments. Though the study in the manuscript is incomplete and inconclusive, it forms a base for future studies with these proteins.
I found the manuscript interesting, and it also projects interesting work. Having said that, there are some concerns about the manuscript, which, when evaluated, in the manuscript and help in future research directions even better. Below are my comments on the manuscript*:

**Response:** We sincerely thank the reviewer for their thoughtful evaluation and valuable suggestions. The feedback has been instrumental in enhancing the clarity, rigor, and overall quality of our manuscript. Below, we provide detailed, point-by-point responses to each of the comments raised.

Please note that the corrected line numbers mentioned in our responses here correspond to the lines marked using the "**Simple Markup"** view under the **"Review"** tab in the revised manuscript. This helps maintain readability while still indicating where revisions have occurred. If this setting is not enabled, you can simply go to the Review tab, locate the Markup section, and in the first drop-down, **Display for Review,** select **“Simple Markup”** (for Microsoft Word 2021).

1. *In the introduction part, it would help to provide some information about the effectors of Gαo proteins. The authors have abruptly jumped into discussing effector proteins*.

**Response:** We thank the reviewer for the insightful comment. In the revised manuscript, we have enlisted some proteins that have been characterized as the effector molecules of Gα_o_ in other model systems, as discussed in the literature.

**Changes made in the manuscript:** Added lines 76 to 78 (known effectors PINS, Rap1GAP, RGS-17, and Rab-5), and added reference [13] with subsequent change in reference numbers. Added lines 105 to 107 (GPRIN-1 and Necdin).

2. *The authors have mentioned SNARE proteins. Nowhere in the manuscript have they described the abbreviation and its significance.*

**Response:** We thank the reviewer for pointing this out. In the revised manuscript, we have included the abbreviation and the significance of SNARE proteins.

**Changes made in the manuscript:**  Added lines 70 to 73

3. *The authors say they prepared brain lysates multiple times to get 12 mg/ml. The scientific way of saying this would be to quantitate it by saying how many brain samples.*

**Response:** We are sorry for not mentioning the number of replicates. In the revised manuscript, we have mentioned that the experiment was performed three times, using four mice in an independent replicate.

**Changes made in the manuscript:** Added lines 153 and 154.

*There are a couple of abbreviations for IP. The authors should either use the full form for one of them or name the buffer with some other acronym.*

**Response:** We are sorry for the confusion caused**.** We have mentioned the full form for immunoprecipitation.

**Changes made in the manuscript:** Changes in line 156.

4. *The manuscript in general has errors/typos that need to be corrected. Also, the units should be written in a consistent manner throughout the manuscript like DL, mM, DM etc.*

**Response:** We apologize for the errors and typos used in the manuscript. We have thoroughly reviewed the entire manuscript and made the necessary corrections accordingly.

**Changes made in the manuscript:** Changes at all places where applicable.

5. *For SEC, the authors have mentioned the specific fraction groups collected but never mentioned the rationale of it. The better way of saying this would be all the fractions were collected.*

**Response:** We thank the reviewer for pointing this out precisely. We have made the necessary changes by mentioning that we have collected all the fractions.

**Changes made in the manuscript:** Added line 213

6. *The authors have mentioned GTPγS as a non-hydrolysable analog of GTP. However, it is a slowly hydrolyzing analog. For this study, better studies would have been other non-hydrolysable analogs such as GMPPCP/GMPPNP*.

**Response**: We sincerely thank the reviewer for this thoughtful and important comment. We agree that GTPγS is more accurately described as a *slowly hydrolysable* analog of GTP. As the reviewer correctly notes, in some experimental contexts, slowly hydrolyzable analogs such as GTPγS may not fully prevent hydrolysis; alternatives like GMPPCP or GMPPNP can be more suitable. In our study, however, as shown in Figures 1B and 1C, GTPγS treatment resulted in robust and sustained activation of Gα_o_ protein throughout the experimental time frame. These data suggest that, under our specific conditions, GTPγS was effective in achieving near-complete Gα_o_ activation, with minimal apparent hydrolysis during the assay. To address the reviewer’s suggestion, we have revised the manuscript to describe GTPγS as a "slowly hydrolysable" analog rather than "non-hydrolysable".

**Changes made in the manuscript:**  Changes made in lines 36 and 244.

7. *An alternative method to check the loading control would have been HPLC, which would be more quantitative and unbiased.*

**Response:** We thank the reviewer for suggesting HPLC as an alternative method to evaluate loading controls and provide a quantitative and unbiased measurement of protein and nucleotide loading. We agree that HPLC can offer precise quantification, particularly of bound nucleotides or total protein levels in purified fractions. In this study, however, our primary objective was to examine Gα₀-interacting proteins under defined nucleotide conditions (GDP vs. GTPγS) using co-immunoprecipitation and mass spectrometry. To assess the consistency of Gα₀ pulldown across conditions, we used Gα₀ western blot normalization in Panels D and F, and further validated equal representation of Gα₀ in both conditions using mass spectrometry peptide counts (Fig. 1G). These collective approaches provided us with both semi-quantitative (western blot) and peptide-level (MS-based) confidence in Gα₀ loading.

**Changes made in the manuscript:**  Added revised Fig. 1 with panels D and F and added lines 466 to 469 (figure legend- Revised Fig. 1, Panel D) and lines 471 to 472 (figure legend- Revised Fig. 1, Panel F).

8. *In the western blot of fig.1, the loading controls are absent, which is standard for the blot images*

**Response:** We thank the reviewer for pointing out the importance of including loading controls in western blot analyses. While traditional housekeeping protein blots are not shown, we addressed sample loading consistency through quantitative normalization of the immunoprecipitated signals, as presented in Revised Fig. 1 Panels D and F. In Panel D, the intensity of Gβ co-immunoprecipitated with Gα₀ was quantified and normalized to the amount of Gα₀ pulled down, ensuring that the observed decrease in Gβ binding in the GTPγS condition is not due to unequal Gα₀ loading, but reflects true biochemical changes upon activation. Similarly, Panel F shows quantification of GPRIN1 interaction with Gα₀, again normalized to Gα₀ levels to confirm that the differences in signal are due to changes in protein-protein interaction rather than loading discrepancies. Together, these quantitative data validate the western blot observations and support the reproducibility of the experimental results despite the absence of traditional loading controls.

**Changes made in the manuscript:**  Added revised Fig. 1 with panels D and F and added lines 466 to 469 (figure legend- Revised Fig. 1, Panel D) and lines 471 to 472 (figure legend- Revised Fig. 1, Panel F).

9. *In the blot of fig 1C, the Gαo bands should be identical, as per the hypothesis and explanations in the text. Nothing has been discussed about why you see a difference in the intensity of Gαo in the GDP and GTPγS blots.*

**Response:** We thank the reviewer for raising this concern. First, the conformational change induced by GTPγS binding may increase the accessibility of the Gα_o_ epitope recognized by the antibody used in the immunoblot, leading to more efficient detection. Also, variation in sample loading or membrane transfer could also contribute to apparent differences. However, regardless of the cause, we believe that this small difference does not alter the core interpretation of the experiment. Quantification of Gβ binding (Fig. 1D) was normalized to the Gα_o_ signal within each condition, ensuring that conclusions regarding activation-dependent dissociation remain valid. Moreover, mass spectrometry (Fig. 1G) confirms comparable recovery of Gα_o_ peptides in both nucleotide states, supporting the view that immunoprecipitation efficiency was not meaningfully different between GDP and GTPγS conditions.

**Changes made in the manuscript:**  Added revised Fig. 1 with panels D and G and lines 466 to 469 (figure legend - Revised Fig. 1, Panel D) and lines 472 to 476 (figure legend- Revised Fig. 1, Panel G).

10. *The author says mass spectrometry could be used to check the effector-binding. A lane in the SDS-PAGE with crude extract, which is not modified to load any nucleotide, would be an interesting sample to compare.*

**Response:** We thank the reviewer for the suggestion. We have included the mass spectrometry data in the revised version.

**Changes made in the manuscript:**  Added Lines 216-235 (Methods- Mass spectrometry protocol), Lines 296 to 307 (Results -mass spectrometry), revised Fig.1 Panel G, and added lines 472 to 476 (figure legend- Revised Fig. 1, Panel G).

11. *In Fig. 2, it is interesting and confusing at the same time, as to how there is a huge fraction from 37–45 which absorbs at 280, but does not read in Coomassie. It would be of interest to get the MALDI done for these samples and check the possibility. That may also give a conclusion to the manuscript.*

**Response:** We thank the reviewer for the suggestion. We did not understand the comment completely. Based on our understanding, we believe that the reviewer wants to talk about Fig.1 and not Fig.2. Regarding the suggestion to use MALDI-MS, we fully agree that mass spectrometry would be informative to identify the constituents of this region. However, for our specific experimental context, i.e., complex immunoprecipitated samples from brain lysates containing potentially low-abundance effectors of Gα_o_, we believe LC-MS/MS is more suitable and informative than MALDI-TOF, offering several advantages like greater sensitivity and dynamic range, better compatibility with in-gel digested samples. (Aebersold et al., 2003, *Nature* **(**https://doi.org/10.1038/nature01511). Indeed, as shown in Fig. 1G, our LC-MS/MS analysis has already revealed differential presence of known Gα_o_ binding partners and candidate effectors, such as GPRIN1, further validating the utility of this approach. Nonetheless, we appreciate the reviewer’s suggestion and recognize that MALDI-MS could serve as a complementary method in future experiments.

**Changes made in the manuscript:**  Added Lines 216-235 (Methods- Mass spectrometry protocol), Lines 296 to 307 (Results -mass spectrometry), revised Fig.1 Panel G, and added lines 472 to 476 (figure legend- Revised Fig. 1, Panel G).
